# Is dry eye disease the same in young and old patients? A narrative review of the literature

**DOI:** 10.1186/s12886-022-02269-2

**Published:** 2022-02-22

**Authors:** Stefano Barabino

**Affiliations:** Ocular Surface & Dry Eye Center, ASST Fatebenefratelli-Sacco, Ospedale L. Sacco, Università di Milano, via GB Grassi, 57, 20157 Milan, Italy

**Keywords:** Dry eye disease, Ocular surface, Tear film, Blink

## Abstract

Advanced age is one of the most evident risk factors for dry eye disease (DED), with male/female sex, chronic drug consumption, and prolonged device use. This article aims to review the literature about the changes of the ocular surface associated with DED in the elderly and patients < 40 years. The pathophysiologic changes of the ocular surface responsible for eye dryness are linked with inflammation and neurosensory abnormalities and may occur with a different feature in young patients compared with elders. Peculiar treatment strategies may be needed for young and older subjects with DED.

## Background

Dry eye disease (DED) is “a multifactorial disease of the ocular surface characterized by a loss of homeostasis of the tear film, and accompanied by ocular symptoms, in which tear film instability and hyperosmolarity, ocular surface inflammation and damage, and neurosensory abnormalities play an etiological role,” as it has been defined by the second report of the Tear Film and Ocular Surface Society, TFOS DEWS II [1]. An aqueous deficient type and an evaporative DED are described due to the different predominant causes of eye dryness; excessive evaporation is the main pathophysiologic mechanism in over 75% of cases, while insufficient tear production is less frequent [2, 3].

DED is associated with several recognized risk factors, among which male/female sex and advanced age are the most evident ones [4]. Symptomatic dry eye prevalence has been reported to increase progressively with aging, independently from sex; however, females are generally affected more often than males, especially after menopause [4, 5]. Overall, signs of eye dryness were reported in 5–30% of the elderly subjects, and the frequency of dry eye was 8.4% for those < 60 years, 15% between 70 and 79 years, and 20% for patients over 80 years [6]. Several factors contribute to induce DED in older adults: increasing rates of systemic and topical drugs, lid laxity, hormonal changes, including menopause, chronic inflammation and oxidative stress [7, 8]. Nevertheless, dry eye is observed at any age, and incidence in young people is increasing [9]. One factor could be the prolonged persistence in front of digital displays due to smart working, e-learning and recreational use, which has become a very common occurrence, especially before 60 years [10]. An observational study investigated the features of the dry eye of patients aged 20–41 years compared to older ones, and found some differences [11].

This article aims to review the literature on dye eye to gather insight into possible differences between patients younger than 40 years and those older than 60 years.

## Methods

For this review of the literature, a non-systematic search was performed in PubMed using the following keywords to retrieve preclinical data: “cornea,” “epithelium,” “UV irradiation,” “oxidative” and “inflammation”. The following keywords were searched to retrieve clinical data: “ocular surface” and “dry eye”. Articles in English or with English abstracts were retrieved. All clinical trials were read and included if the age of patients was a parameter, while reviews and preclinical evidence were chosen based on the Authors’ evaluation of relevance. The search concluded in March 2021, and included all articles present in PubMed, without temporal limits.

### Dry eye disease in different ages

Recently, special characteristics were observed in the tear film lipid layer of young patients with DED compared to older patients [11]. In a retrospective study on 675 patients with DED, three age groups were compared (20–41 years, 41–60 years and > 60 years). More severe subjective symptoms and more incomplete blinks were found in the younger subgroup, and were explained by higher corneal nerve density and lower pain threshold. Patients in the younger group also had a lower mean lipid layer thickness, which was negatively correlated with the Standardized Patient Evaluation of Eye Dryness (SPEED) symptom score (r = − 0.136, *p* < 0.001). Conversely, the mean number of functioning meibomian glands was higher and glandular loss was less than in the other age groups. Younger patients had more incomplete and total blinks (both *p* < 0.001) and a significantly higher incomplete blink rate (*p* = 0.006) compared with the other groups. In conclusion, in young patients, DED is characterized by the reduction of the lipid layer, and clinically by severe symptoms and impaired blinking; on the contrary meibomian number and function are preserved. It is possible that the thin lipid layer provides weak protection against tear evaporation and may cause severe symptoms and more blinks; incomplete blinks may be inefficient for squeezing of the meibomian glands by the orbicularis muscle, resulting in a lower meibum secretion [11]. Impairment of blinking in patients with DED had previously been demonstrated by Su et al. [12]; using blinking recording by a high-speed camera, short blink intervals and prominent incomplete blinks were found.

Elderly patients (< 60 years) with DED have been found to have milder symptoms compared to younger patients under 41 years, although older subjects have a lower number of functioning Meibomian glands and a greater gland loss [11]. On the contrary, altered blinking is less frequent in older patients. In addition, in elders, involutional eyelid malposition is frequent, and is mainly associated with horizontal lid laxity. Eyelid malposition, in turn, leads to corneal exposure, poor tear film distribution, and abnormal tear outflow, which together induce eye dryness [11].

### Risk factors for dry eye and age

According to an expert consensus, a DED diagnosis encompasses the presence of unstable tear film, ocular surface inflammation, epitheliopathy and neurosensory abnormalities [13]. It is known that the integrity of the ocular surface is necessary for good vision, and is preserved by the balanced and correct activity of neural, hormonal and immunological mechanisms [14]. Several mechanisms have a role in the physiopathology of dry eye. They may have different importance according to the frequency of some risk factors in different ages of life. Autoimmunity involves Th1 and Th17 cell responses, IL-17A, IFN-γ, and GM-CSF secretion [15–17]. The release of inflammatory factors reduces the secretion of tears and mucin, increases the instability of the tear film, promotes the evaporation of tears, and finally causes the hyperosmotic environment of the eye surface [18, 19]. Conjunctival cells seem to be more vulnerable to stimuli than corneal cells, probably due to the higher presence of local immunity and increased risk of inflammation [20].

#### Factors inducing dry eye, easily associated with people under 40 years

Some factors that may promote dry eye occurrence, although pertaining to any age, are more often found in young people than in subjects older than 60 years. In the last decade, digital display use has become ubiquitous, especially for young people, and the current COVID-19 outbreak has made screen use even more essential to work and everyday life. Unfortunately, prolonged persistence in front of digital displays has been a contributing factor for DED due to several mechanisms, and increased prevalence of dry eye signs and symptoms was found in digital display users. Abnormal blinking during computer operation, including a reduced blink rate and incomplete eyelid closure, high visualization angles leading to increased palpebral fissure, and dysfunction of meibomian glands, were associated with long-term display use and may contribute to compromising the ocular surface integrity [10]. In addition, significant reductions in tear volume and stability, altered composition and increased osmolarity of the tear film, expression of inflammatory cytokines, increased concentration of oxidative stress markers, reduced mucin secretion, eyelid abnormalities, encompassing corneal and conjunctival staining and bulbar redness, were described as a direct consequence of digital display use [10]. Data from an experimental study on an animal model showed that eye dryness was associated with a reduction of the blinking frequency, which is similar to the results reported by screen users. In addition, the histopathologic changes observed in challenged mice suggested the involvement of lacrimal hypofunction. A decrease in tear secretion was accompanied by a decrease in the acinar cell number, and residual acinar cells were enlarged, had an increased volume of secretory vesicles, and a loss of intracellular cell structure [21].

In clinical practice, patients who use contact lenses, and those working in inappropriate environments, are mainly prone to be damaged by prolonged use of displays [10]. The clinical manifestations of prolonged smartphone use in 80 healthy volunteers were subjective symptoms of DED, tear film instability and increased oxidative stress indices in tears and at the ocular surface. Oxidative stress markers in the tear film evaluated in this study included hexanoyl lysine, 4-hydroxy-2-nonenal, malondialdehyde and 8-oxo-2′-deoxyguanosine, and were measured using ELISA. Reactive oxygen species at the ocular surface were measured through 2′,7′-dichloro-dihydrofluorescein diacetate [22].

Some difference exists in the offending potential of screen devices. A clinical study carried out in 31 healthy subjects aged 20–26 years compared the effect on the ocular surface of different screen devices. Smartphones and e-readers were found to be less detrimental in comparison with laptops, computers and tablets (*p* < 0.05), as assessed based on Ocular Surface Disease Index, Computer Vision the Syndrome Questionnaire, tear meniscus height, the Schirmer I test, noninvasive keratograph break-up time, osmolarity and bulbar redness [9].

It was suggested that dry eye could be promoted by overexposure to blue light with short wavelengths (produced by screen devices) by increasing the production rate of O2•−, the expression of inflammatory factors in the cornea and tear film and decreasing cell viability [20, 23].

Sunlight exposure, which may be increased by outdoor professional or recreational activities, is challenging for the superficial eye tissues, and may promote dry eye [24]. In a three-dimensional corneal epithelial tissue model, non-toxic doses of UV irradiation-induced intracellular ROS accumulation suggest that the oxidative stress linked to light exposure may impact eye surface aging. In addition, it was observed that desiccating conditions produced lipid peroxidation and IL-8 release from tissues, which were limited by the addition of lubricant eye drops [25].

#### Risk factors prominent after 60 years of age

Both aqueous tear deficiency and evaporative dry eye are more frequent in older than in young subjects due to several concurrent factors [26]. In the advanced age, tear production becomes less adequate because of several events, including increased systemic and topical drug use, lid laxity, hormonal changes, such as menopause, systemic inflammatory conditions and a higher prevalence of autoimmune diseases (mainly Sjogren’s syndrome and rheumatoid arthritis). As an example, consumption of at least five drugs is rare before 20 years, occurs in 8% of subjects 20–59 years old, and in 37% after 60 years of age [27]. Abnormalities in eyelid positioning (laxity, floppy eyelid syndrome, retraction and lagophthalmos), meibomian gland dysfunction, rosacea, abnormal corneal sensation, and decreased blink reflex are frequent in elderly subjects. All these factors contribute to making the tear film break-up more rapid [28–30].

Increased tear osmolarity and high levels of inflammatory cytokines have been detected in the tears of dry eye patients [31]. Hyperosmolarity may follow to inadequate aqueous tear film, due either to reduced production or increased evaporation, and induces oxidative stress and inflammation, which increase in aging [32].

Goblet cells produce mucin, which exerts a protective function, clears debris, prevents bacterial adhesion, promotes lubrication and maintain the epithelial barrier function [33]. With aging, up to over 60 years, the number of goblet cells remained unchanged; however, the cell functions declined [34]. The goblet cell number and secretory function are reduced in the presence of chronic inflammation; it has been observed that IFN-gamma and TNF-α induce goblet cell apoptosis [35–37]. Corneal epithelial cells with inadequate mucin protection are left vulnerable to cell damage. Cell damage is further perpetuated by cytokine production, inflammatory response and injured corneal epithelial cells; in addition, conjunctival cells are more prone to apoptosis in the older age [38]. In older adults with dry eye, functional goblet cells are lost, goblet cell apoptosis is increased, and impaired mucin production may favor advanced DED. In subjects with advanced stages of DED, corneal keratinization, corneal ulceration and band keratopathy can be observed [39].

Frequent causes of lid malposition are involutional entropion and involutional ectropion, whose prevalence is 2.1 and 2.9%, respectively, in patients 60 years or older [28]. Patients with malpositioned lids can subsequently develop chronic blepharitis, chronic conjunctivitis, superficial punctate keratopathy from abnormal Meibomian gland secretory function, mechanical injury and cornea exposure. DED occurs in 50–70% of patients with malpositioned lids [28].

Conjunctivochalasis is another known contributor to tear outflow defect and is characterized by redundant bulbar conjunctiva interposed between the globe and the eyelid [40]. The prevalence of conjunctivochalasis increases with age from 71.5% in patients 50 years or younger to greater than 98% in patients > 61 years of age [41].

Systemic and topical drugs contribute to reducing tear production along with aging, predisposing the over-60-year-old population to DED. Systemic medications, including antidepressants, diuretics, dopaminergic drugs and antimetabolites, are commonly used in older patients and cause or exacerbate dry eyes. Drug consumption increases with aging and drug clearance changes as hepatic and renal function decline. A reduced clearance is associated with increased plasma half-life and a higher risk of adverse effects, including eye dryness [42, 43].

The use of ophthalmic medications, such as topical glaucoma medications, is frequent in the elders and is associated with an increased risk of development of dry eye relative to age-matched controls [44]. Glaucoma prevalence and use of more than one topical medication for glaucoma are higher in the elderly than in the younger population [45]. Decreased tear production, with Schirmer’s test results indicating tear deficiency (< 5 mm), has been assessed in 61% of patients using one or more pressure-lowering drops [46]. In a retrospective study, 63% of adults using glaucoma drops had signs and symptoms of DED, starting at a mean age of 55 years. Only 23% of subjects who did not use glaucoma drops developed symptoms and signs of DED, and only after a mean age of 70 years [47]. Since more elderly patients use glaucoma eye drops than the young population, the older adults on glaucoma medications are at an even increased risk of severe dry eye sequelae.

Lacrimal gland dysfunction is another common disease frequently causing aqueous DED in the elderly, especially women. This defect seems to be related to reduced levels of androgens, as the secretory function of the lacrimal gland is known to be regulated by these hormones [48, 49]. Serum levels of dehydroepiandrosterone sulfate are especially low in women with Sjogren’s syndrome, older men and older women [50]. Decreased dehydroepiandrosterone sulphate levels in older men were correlated with dry eye symptoms and insufficient lacrimal gland function, assessed as Schirmer’s test result < 5 mm (*r*= 0.13) [51]. The androgen level is easily diminished below the critical amount needed for optimum eye health in older women, as basal levels are lower in women than the levels in men [52]. In addition, estrogen levels are reduced after menopause, and estrogen is known to stimulate meibomian glands and help regulate ocular surface homeostasis [49].

Roszkowska et al. [53] reported that mechanical sensitivity of peripheral cornea decreases gradually throughout life, whereas central corneal sensitivity remains stable until the age of 60 years and then decreases sharply subsequently. It is usually believed that the reduction in corneal sensitivity developing in aging subjects predisposes older adults to DED [30]. However, the role of corneal sensitivity in dry eye is conflicting. Some studies found decreased sensitivity in patients with dry eyes compared with controls using non-invasive measurements by noncontact esthesiometer [54, 55]. On the contrary, other researchers found that corneal sensitivity was increased in patients with DED; these results could be due to impairment of the epithelial barrier function [56, 57]. However, it is well known that nerves in dry eye patients have the characteristic beadlike transformation, which is thought to represent nerve damage due to inflammatory processes occurring in the presence of DED [58, 59]. Regardless of the direction of change in corneal sensitivity, elderly patients with DED are at higher risk of developing symptoms due to corneal nerve alterations. Both hypersensitivity and hyposensitivity are responsible for clinical discomfort and may induce complications; patients with corneal hypersensitivity experience increased ocular surface discomfort, while those with decreased sensitivity are prone to exposure keratopathy [39].

Lactoferrin content in the tear film was reduced both in aged people and in subjects with DED [60, 61]. In the tear film, lactoferrin has anti-inflammatory, antioxidant and antimicrobic activities. It interacts with the natural and induced immunity interfering with the inflammatory responses [62]. The chelating ability of lactoferrin is responsible for the antioxidant and antimicrobial activities, as it prevents the formation of iron-dependent hydroxyl radicals during inflammatory responses and microbial infections [62, 63]. Indeed, the subtraction of iron by lactoferrin limits bacterial growth and survival because iron is a critical co-factor for bacterial proliferation, as shown in preclinical studies with a substitute for lactoferrin, lactobionic acid [64, 65].

## Conclusion

DED is a debilitating disease occurring in all ages, with increased frequency in older subjects, and especially in advanced age women.

Younger patients may be exposed to risk factors because of working or recreational activities. Patients under 41 years of age often have severe subjective symptoms and incomplete blinks, with a thin lipid layer, but the number of functioning meibomian glands is elevated and glandular loss is limited. In older subjects, glandular loss is a predominant feature, with ocular surface inflammation. These characteristics may be related to the frequent factors producing DED in the elderly, such as systemic inflammatory diseases, glaucoma and topical or systemic drug use.

Although DED treatment is not within the aim of this review article, we suggest that differences between patients of different ages may require peculiar treatment strategies for young and older subjects. As an example, it was suggested that riboflavin supplementation might help maintain the structure and function of the ocular surface, while hormonal therapy, control of excessive use of topical medication, and lactobionic acid may be useful in the elderly [8, 14, 66, 67]. In conclusion, our literature review showed peculiar features of dry eye are linked to age, but the subject is little investigated, and further study is needed.

## Data Availability

Not applicable.

## Abbreviations

DED: Dry eye disease
SPEED: Standardized Patient Evaluation of Eye Dryness
